# Persistence at distributional edges: Columbia spotted frog habitat in the arid Great Basin, USA

**DOI:** 10.1002/ece3.1627

**Published:** 2015-08-18

**Authors:** Robert S Arkle, David S Pilliod

**Affiliations:** U.S. Geological Survey, Forest and Rangeland Ecosystem Science Center970 Lusk Street, Boise, ID, 83706, USA

**Keywords:** American beaver, Amphibian, *Castor canadensis*, habitat association, habitat availability, North America, occupancy, *Rana luteiventris*, species distribution, trout

## Abstract

A common challenge in the conservation of broadly distributed, yet imperiled species is understanding which factors facilitate persistence at distributional edges, locations where populations are often vulnerable to extirpation due to changes in climate, land use, or distributions of other species. For Columbia spotted frogs (*Rana luteiventris*) in the Great Basin (USA), a genetically distinct population segment of conservation concern, we approached this problem by examining (1) landscape-scale habitat availability and distribution, (2) water body-scale habitat associations, and (3) resource management-identified threats to persistence. We found that areas with perennial aquatic habitat and suitable climate are extremely limited in the southern portion of the species’ range. Within these suitable areas, native and non-native predators (trout and American bullfrogs [*Lithobates catesbeianus*]) are widespread and may further limit habitat availability in upper- and lower-elevation areas, respectively. At the water body scale, spotted frog occupancy was associated with deeper sites containing abundant emergent vegetation and nontrout fish species. Streams with American beaver (*Castor canadensis*) frequently had these structural characteristics and were significantly more likely to be occupied than ponds, lakes, streams without beaver, or streams with inactive beaver ponds, highlighting the importance of active manipulation of stream environments by beaver. Native and non-native trout reduced the likelihood of spotted frog occupancy, especially where emergent vegetation cover was sparse. Intensive livestock grazing, low aquatic connectivity, and ephemeral hydroperiods were also negatively associated with spotted frog occupancy. We conclude that persistence of this species at the arid end of its range has been largely facilitated by habitat stability (i.e., permanent hydroperiod), connectivity, predator-free refugia, and a commensalistic interaction with an ecosystem engineer. Beaver-induced changes to habitat quality, stability, and connectivity may increase spotted frog population resistance and resilience to seasonal drought, grazing, non-native predators, and climate change, factors which threaten local or regional persistence.

## Introduction

On the edge of geographic distributions, species often face environmental conditions near the limits of their physiological tolerance. These conditions can challenge individuals’ abilities to resist stressors (e.g., antagonistic non-native species) and exhibit resilience to habitat changes or disturbances (e.g., prolonged drought, wildfire). For example, in western North America, the southernmost populations of species distributed along latitudinal gradients may be especially vulnerable to regional warming and drought effects expected to occur under many climate change scenarios (Gerick et al. 2014). Under these scenarios, aquatic species inhabiting already arid regions may be particularly vulnerable to climatic stressors because of expected changes in the amount or timing of surface water accumulation. Further, the potentially substantial interactive effects of climate change and localized anthropogenic stressors are just beginning to be understood (Ryan et al. 2014).

**Figure 1:**
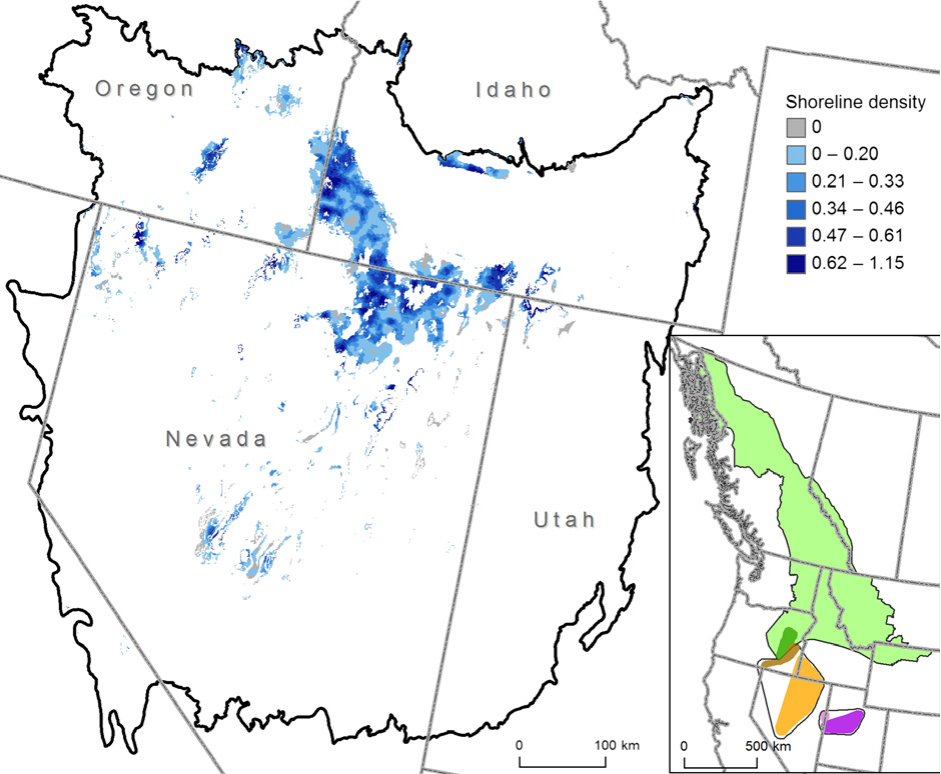
Great Basin study area (black line) and perennial shoreline density (km/km^2^) within areas of suitable climate for Columbia spotted frog breeding. Darker blue pixels (each 270 m) have more km of perennial shoreline within 5 km, whereas gray pixels have no perennial shoreline within 5 km. White areas had <0.20 probability of breeding climate suitability and were masked from shoreline density calculations. Inset shows phylogeography based on mitochondrial DNA analysis (modified from Funk et al. 2008). Three main Columbia spotted frog clades are shown (thin black lines) with color-coded nested clades. Southeast Oregon (dark orange) and southwest Idaho/Nevada (light orange) nested clades belong to the Great Basin Distinct Population Segment (DPS).

One species facing such challenges is the Columbia spotted frog (*Rana luteiventris*), which ranges from southern Yukon, Canada, to central Nevada, USA (Fig.1). This latitudinal gradient covers over 2700 km, among the largest for any amphibian species. At the southern end of this distribution is the Great Basin Distinct Population Segment (DPS). This genetically distinct population is geographically isolated from the large, panmictic northern clade, smaller neighboring clades to the east, and the closely related Oregon spotted frog (*Rana pretiosa*) to the west (Funk et al. 2008). Genetic isolation and speciation (in the case of *R. pretiosa*) resulted from changes in climate and habitat during the Pleistocene glaciation cycles that created relatively large areas of inhospitable environmental conditions, mainly in the southern portion of the range (Bos and Sites 2001; Funk et al. 2008). The most recent glacial cycle reached a maximum about 23,000 years ago and was coincident with cooler, more mesic conditions in the Great Basin- conditions that probably made lower-elevation sites more suitable for Columbia spotted frogs. Molecular evidence suggests a recent northward expansion of the species and a contraction and diversification in the southern part of its range (i.e., the Great Basin; Funk et al. 2008). As the Great Basin has become warmer and drier following the last glacial maximum, populations in this portion of the range have become isolated (usually at mid-elevations on regional mountain ranges) and genetically distinct (Funk et al. 2008; Fig.1).

In addition to adverse climatic conditions, Great Basin populations of the Columbia spotted frog have been exposed to almost two centuries of anthropogenic habitat alterations, beginning with American beaver (*Castor canadensis*) trapping in the 1820s. To de-incentivize American settlement of Great Britain's Pacific Northwest Territories, beaver populations were intentionally extirpated in much of the region (Clements 1991). Removing beaver from arid and semiarid landscapes likely caused changes in channel morphology and groundwater processes, reduced perennial wetland habitats, and altered riparian vegetation (Gibson and Olden 2014). Many studies have found positive associations between frog species and beaver ponded stream habitat (Cunningham et al. 2007; Karraker and Gibbs 2009; Popescu and Gibbs 2009; Hossack et al. 2015), but this association is expected to be especially important where permanent lentic habitats are rare and required for breeding, as is the case with the Columbia spotted frog in the Great Basin.

The next wave of habitat alterations came with American settlement of the region and associated development of water resources for homesteads, livestock grazing, and farming. Much of this settlement occurred near permanent water sources and in valley bottoms, likely affecting spotted frog habitat quantity and quality. Non-native aquatic predators (e.g., brook trout [*Salvelinus fontinalis*], brown trout [*Salmo trutta*], American bullfrog [*Lithobates catesbeianus*]), translocated native aquatic predators (e.g., redband trout [*Oncorhynchus mykiss* ssp.], Lahontan cutthroat trout [*O. clarkii henshawi*]), and novel pathogens (e.g., the amphibian chytrid fungus [*Batrachochytrium dendrobatidis*]) followed human development of the region as these species were intentionally or inadvertently introduced into the region's waterways. These organisms can have negative effects on spotted frog populations, even in otherwise high-quality habitats (Pilliod and Peterson 2001; Pearl et al. 2004, 2009c; Wente et al. 2005; Adams et al. 2010; Pilliod et al. 2010b).

The small, isolated Columbia spotted frog populations in the Great Basin were deemed highly susceptible to extinction due, in large part, to degradation and fragmentation of habitat. This DPS has been a candidate for federal protection under the U.S. Endangered Species Act since 1993 (U.S. Fish and Wildlife Service, 2013). The goal of this study was to use habitat modeling and analyses of potential threats to better understand factors facilitating persistence of populations at distributional edges. To this end, we used existing data on Columbia spotted frogs to determine the following: (1) landscape-scale (i.e., Great Basin-wide) habitat availability and distribution; (2) water body-scale habitat associations; and (3) the strength of evidence for hypothesized threats to persistence.

## Methods

### Study area

The Great Basin is a 40-million-ha region of the western United States dominated by arid and semiarid grasslands, shrublands, and piñon–juniper woodlands, with conifer species at higher elevations (Fig.1). Our study area was defined by the boundaries of three level III ecoregions (Snake River Plain, Northern Basin and Range, and Central Basin and Range; U.S. Environmental Protection Agency). Precipitation in this region varies strongly along elevation gradients and falls mainly as winter snow and early spring rain. Summers are hot and dry throughout the region. Aquatic habitats are limited in much of the region and consist mainly of small streams, ephemeral ponds, and stock ponds. Most of the data used in our study were derived from surveys of public land.

### Habitat availability

To assess the availability and spatial distribution of potentially suitable habitat (Question 1), we extracted spatial information on all perennial streams, rivers, canals, connectors, lakes, ponds, and reservoirs within the study area from NHDPlus version 2.1 (U.S. Environmental Protection Agency and U.S. Geological Survey 2012). Ephemeral water bodies were not used because they are unlikely to support reproduction in this region and because all life stages are aquatic obligates. We merged and converted all features to lines and ran a line density analysis (ArcGIS 10.2 software; ESRI, Redlands, CA, USA), calculating the density of perennial shoreline within a 5-km moving window (maximum spotted frog dispersal distance; Reaser 1996) for each 270-m pixel in the Great Basin. We then clipped the resulting shoreline density raster to areas with climate suitability ≥0.20, according to a maximum entropy model (10-fold AUC average ± SE = 0.95 ± 0.006; Pilliod et al., in press) relating monthly, 30-year average temperature and precipitation values to spotted frog occupancy at 145 spotted frog breeding sites in the Great Basin. The 0.20 climate suitability cutoff value was selected because the resulting area contained >95% of all breeding observations made for this relatively rare species since 1993. Using a kappa or TSS thresholding approach would not guarantee that >95% of all sites were included in the clipped area, while the high model AUC suggests that the false-positive rate is sufficiently low that overpredicting to truly unoccupied areas would be unlikely. The output raster represents perennial shoreline habitat density within areas having climates similar to those suitable for breeding. The output does not indicate habitat quality at the water body scale, but represents catchment- (i.e., HUC-12) or regional-scale quality and quantity. We assessed the distribution (numeric, not spatial) of shoreline density values within this “Available” habitat and separately calculated the percentage of available habitat belonging to different landowners (potentially important for conservation management) by creating probability density distributions using the “sm” package in R Studio 0.98.507.

### Water body-scale habitat associations

Empirical data on water body-scale (i.e., individual ponds, lakes, or distinct stream segments, such as an oxbow, pool, beaver pond, or riffle) spotted frog occupancy and habitat parameters were collected between 2001 and 2012 in Idaho, Nevada, and Oregon as parts of separate studies by state or federal agencies and other researchers (Table1; see Appendix 1 for variables collected). We used these data to address Questions 2 and 3. In each of these studies, catchments (HUC-12 scale) were selected for surveying of water bodies either randomly within the known range of the DPS, or as repeat visits to historically occupied catchments. Within each catchment, visual encounter and dip net surveys were conducted during summer months to determine presence of spotted frogs, by life stage, in every known water body (i.e., sample unit). A multiple-observer protocol was used to search along shorelines and vegetated areas for amphibians which were captured, identified to species, quantified by life stage, and released. This approach can result in high detection probabilities for spotted frogs when they are present (Wente et al. 2005). Although the majority of our sites were visited on one occasion, the 65 Idaho sites visited >1 time had a mean detection probability of 0.89. Consequently, we used naïve occupancy rates in our analyses. Field crews also quantified habitat parameters and occurrences of other aquatic species (Appendix 1; see Appendix 8 for fish species encountered). Each study used slightly different protocols to collect or quantify habitat variables (Appendix 1). After attempting to combine the three datasets (i.e., by reformatting similar variables and dropping variables not shared by the three datasets) to conduct a single global analysis, there was an insufficient number of variables to provide a meaningful view of habitat preferences and threats. Further, land management (e.g., grazing practices, wetland restoration), available habitat types (e.g., beaver ponds; Appendix 2), spotted frog prevalence (Table1), and sample sizes (Table1) varied substantially among datasets, and ignoring these inherent subsets in the data could obfuscate regional differences in habitat associations. Conducting separate analyses for each state had the benefits of informing land managers of habitat associations at a more local scale, while providing an assessment of the generality of results by comparing across states. As indicators of spotted frog breeding (i.e., egg masses or larvae) were not frequently detected, we modeled occupancy of any life stage of the species.

**Table 1:** Sample sizes and naïve occupancy rates for each subset of data used in analyses of Columbia spotted frog habitat availability, water body-scale occupancy, and threats to persistence

Analysis	Data subset	Sample size	Naïve Occupancy rate	Data source^1^
Habitat availability	Spotted frog breeding locations	145	1.0	USFWS
Water body-scale habitat associations^2^	ID^3^survey sites	78	0.28	IDFG
	NV^3^survey sites	778	0.14	NDOW, USFS
	OR^3^survey sites	75	0.15	Wente et al. (2005)
Threats to persistence^4^	ID survey sites	78	0.28	IDFG
	NV survey sites	1,129	0.12	NDOW, USFS
	OR survey sites	96	0.15	Wente et al. (2004)
	Bullfrog GIS observations	182	1.0	USFWS, USGS NAS, USGS BISON, Wente et al. (2004)
	Redband stream vertices^5^	111,561	1.0	(May et al. 2012)
	Cutthroat stream vertices^5^	11,428	1.0	USFWS
	Available 270-m pixels^6^	412,634	na	Pilliod et al. (in press)
	Chytrid observations	32	0.47	(Olson et al. 2013), USFWS

1 Data source abbreviations are as follows: USFWS: U.S. Fish and Wildlife Service; IDFG: Idaho Department of Fish and Game; NDOW: Nevada Department of Wildlife; USFS: U.S. Forest Service; USGS NAS: U.S. Geological Survey Nonindigenous Aquatic Species program; and USGS BISON: USGS Biodiversity Information Serving Our Nation. BISON records occurring at county centroids were excluded to prevent artificial inflation of bullfrog distributions.

2 For occupancy modeling, only sites with no missing habitat data were used.

3 State abbreviations are as follows: ID: Idaho; NV: Nevada; and OR: Oregon.

4 For threat analyses, all sites where a given variable was recorded were used in analyses of that variable (i.e., includes sites missing other habitat data). Consequently, sample sizes vary with threat variable analyzed. Values shown are maximum possible sample sizes.

5 Redband and cutthroat trout vertices were derived from GIS data of streams segments categorized as occupied by each fish species. Here, each vertex is assumed to be occupied.

6 Available pixels are those with ≥0.20 probability of having suitable climate for spotted frog breeding.

To determine water body-scale habitat associations (Question 2), we used habitat variables to predict occupancy with nonparametric multiplicative regression (NPMR) in HyperNiche 2.22 (McCune and Mefford 2009). This approach to occupancy modeling allowed us to investigate how habitat parameters interact with one another in nonlinear, multiplicative ways to predict occupancy from presence–absence field survey data (McCune 2006). NPMR has been used in similar analyses (e.g., Grundel and Pavlovic 2007; Fenton and Bergeron 2008; Welsh and Hodgson 2011; Pilliod et al. 2013; Arkle et al. 2014) and it is well suited for modeling relationships between species and their habitats because: (1) species’ responses to environmental gradients have long been conceptualized as being nonlinear (e.g., Gaussian or more complex [Whittaker 1956;]) in form, and (2) there is wide acceptance that both a species’ niche (Hutchinson 1957) and its ability to occupy an area (Shelford 1931) is defined by an individual's simultaneous response to many interacting environmental factors (i.e., Shelford's law of tolerance), with occupancy precluded by a lack of suitability of any one factor (McCune 2006). NPMR accommodates these concerns by optimizing the fit of data to a local model (as opposed to a fixed linear, quadratic, or logistic “global” model), where the occupancy rate of a species in an environmental neighborhood (the multidimensional combination of values for all habitat variables) is used to estimate the probability of occupancy at nearby (in multidimensional environmental space) “target” points (McCune 2009). Kernel smoothing functions are used to more heavily weight observations nearer to the target point. In this manner, a flexible, nonlinear response curve is generated based on each combination of predictor variable values (i.e., multiplicative interactions) in a given model. To select the best model for a given analysis, all combinations of habitat variables and their smoothing functions are evaluated and we defined the best fitting model as that which resulted in a ≥ 2.5% increase in fit (assessed with log likelihood ratio, log*β*; Arkle et al. 2014) over the next-best model with one less predictor variable.

In each NPMR analysis, we used a local mean model (LLM) and Gaussian weighting functions to conduct free-search iterations of combinations of predictors (screened to remove correlated variables) and their tolerances (tolerance = SD of Gaussian weighting function of each predictor) to maximize model fit (assessed with log likelihood log*β*), while minimizing overfitting (see McCune 2009, for details on these, and subsequent, model parameters). Log*β* evaluates the improvement of the fitted model over the naïve model (i.e., the overall occupancy rate), expressed in powers of 10. We controlled overfitting through minimum average neighborhood size, minimum data-to-predictor ratio, and “leave-one-out” cross-validation. As log*β* is calculated using this cross-validation technique, the error rate in model development data is expected to approximate that of validation datasets. Consequently, we used full datasets, rather than withholding validation data, to maximize our ability to detect trends in occupancy. Bootstrap resampling (each dataset resampled with replacement 100 times) was used to quantify the stability of models (when different combinations of water bodies were analyzed) by providing an average log*β* (±SE). We also report the average neighborhood size (N* = average number of sample units contributing to the estimate of occupancy at each point on the modeled surface) and Monte Carlo randomization results (null hypothesis = fit of best model is no better than chance, using the same number of predictor variables in 100 free-search iterations with randomly shuffled response values). For each predictor variable in each final model, we report tolerance (a measure of niche breadth) and sensitivity (a measure of relative importance of quantitative predictors, not the “true positive rate” *sensu* species distribution modeling). High tolerance values, relative to the range of the predictor, indicate that data points farther in predictor space are used to estimate response values at the target point. Sensitivity, which indicates the relative importance of quantitative predictors, is scaled from 0 to 1 where a value of 1 indicates that, on average, changing the value of the predictor by ±5% of its range results in a 5% change in the response estimate. This provides an estimate of relative importance for each quantitative predictor in the model.

### Threats to persistence

To evaluate potential threats to persistence (Question 3), we related habitat variables identified by resource managers (U.S. Fish and Wildlife 2013) to spotted frog occupancy in each state (Table2). Although variables representing the different threats were included as potential predictors in habitat-association models described above (and some were included in final habitat-association models), many of the threat-associated variables were correlated with other habitat variables (e.g., hydroperiod permanence was related to maximum water depth), which tended to preclude them from inclusion in final habitat-association models. As each threat was specifically hypothesized to be important to regional persistence, we viewed them as worthy of individual examination for understanding factors associated with persistence at the edge of this species’ distribution. Consequently, for each state, threat, and value of a given categorical variable, we calculated the following: (1) the naïve occupancy rate (to represent habitat use relative to availability) and (2) the proportion of occupied sites attributed to that categorical value (to determine locations where spotted frogs tend to occur in the current landscape, regardless of availability or survey effort for the given categorical value). Categories of a particular variable with high or low occupancy rate and high or low proportion of occupied sites provided strength of evidence for a particular hypothesized threat. We used data from field surveys to examine each threat except for those related to bullfrogs and the amphibian chytrid fungus (*Batrachochytrium dendrobatidis* – Bd).

**Table 2:** Threats to Columbia spotted frog persistence identified by resource managers and summary of findings from this or other studies

Threat category	Threat	Habitat variables examined	Effect on/corr. with spotted frog occupancy	Data source/citation
Habitat loss	Beaver habitat loss	HABITAT	negative	Fig.3, Table3, Appendix 2
	Fragmentation	WATERCON, LOTICLENTIC/WATERPERM	negative	Appendix 5, Hossack et al. (2015), Pilliod et al. (in press)
	Grazing	GRAZEIMPACT GRAZED	negative	Appendix 6, Appendix 7, Pilliod and Scherer (2015)
Predation/disease	Salmonids	FISHSTATUS	negative	Fig.2, Fig.4, Appendix 8, Appendix 9
	Centrarchids	FISHSTATUS	nd	More surveys needed
	Bullfrogs	Location data	na	Fig.2, Appendices 9, 10, 11, Pearl et al. (2004); Monello et al. (2006)
	Amphibian chytrid fungus	Location data	na	Appendices 10b and 11, Van Horne (2012)
Climate/weather	Climate change	HYDROPERIOD	negative	Appendix 12, Pilliod et al. (in press)
	Drought	HYDROPERIOD	negative	Appendix 12

na = threat was not examined using data from habitat surveys; occurrences from GIS databases were used instead (see Table1).

nd = none detected during habitat surveys.

In addition, we used occurrence data on predatory and pathogenic species from GIS databases (Table1) to better understand the extent and conditions under which spotted frog habitats are used by these species throughout the Great Basin. We compared the distributions of spotted frog breeding locations, bullfrog observations, and native trout-occupied stream segments across gradients of shoreline density and climate conditions (30-year [1981–2010] average temperature and precipitation values) using probability density distributions for each species (see Table1 for sample sizes and data sources). We also evaluated the spatial distribution of Bd and the proximity of this pathogen to spotted frog breeding sites. Because bullfrogs and Bd were not prevalent at the particular sites where habitat and spotted frog occupancy data were collected, we could not include them in our habitat-association models or in our threats to persistence analyses using field data. Instead, we focused our analyses of these two potential threats on their distributions and proximities to spotted frog breeding sites rather than on evaluating the magnitude of threat posed (i.e., effects on occupancy probability).

## Results

### Habitat availability

**Figure 2:**
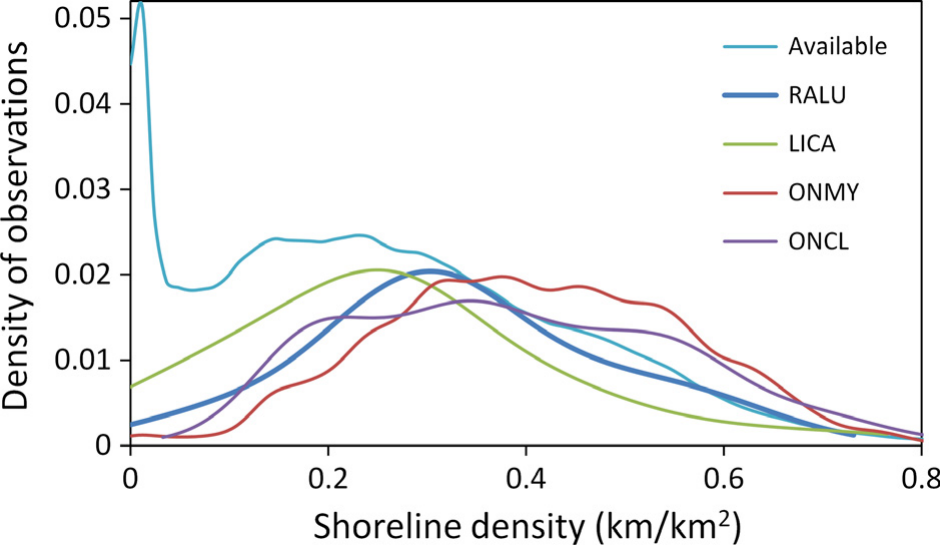
Kernel smoothed probability density of shoreline density values within areas of the Great Basin having suitable climate for Columbia spotted frog breeding (“Available”) and at locations of species observations. Shoreline density was calculated using a 5-km moving window around each 270-m pixel. Sample sizes for distribution calculations are provided in Table1. Mean ± SE shoreline density values are as follows: 0.33 ± 0.01 (spotted frog; RALU), 0.26 ± 0.02 km/km^2^ (bullfrog; LICA), 0.41 ± 0.0005 (redband trout; ONMY), and 0.38 ± 0.001 (Lahontan cutthroat trout; ONCL). Mean values are 21% lower, 18% greater, and 13% greater (respectively) than the mean spotted frog breeding site value.

Only 5.4% of the 40-million-ha Great Basin study area had at least some perennial water within 5 km and climate suitability ≥0.20 (Figs.1 and 2). Over 80% of these suitable hectares were contained in 14 (of 1,586) large patches of contiguous suitable areas, with the largest patch alone containing 60% of suitable hectares. Only nine patches were known to be occupied and they tended to be large (5 of 9 occupied patches were in the largest 2% of patches). However, many large suitable patches have no record of spotted frog occupancy (i.e., of the 14 largest patches, only three were known to be occupied). Within areas of suitable climate, perennial shoreline habitat was limited, with 10% of the climatically suitable area having 0 km shoreline/km^2^ (see “Available” data series Fig.2).

Fifty-six percent of the area with suitable climate conditions occurred on lands managed by the U.S. Department of Interior's Bureau of Land Management (BLM), whereas only 21% and 15% occurred on private and U.S. Department of Agriculture Forest Service (USFS) lands, respectively. BLM lands, however, tended to have the lowest shoreline densities (Appendix 3), whereas USFS lands contained the majority of areas with high shoreline densities. State and private lands tended to contain areas of intermediate shoreline densities (relative to BLM and USFS).

### Water body-scale habitat associations

**Figure 3:**
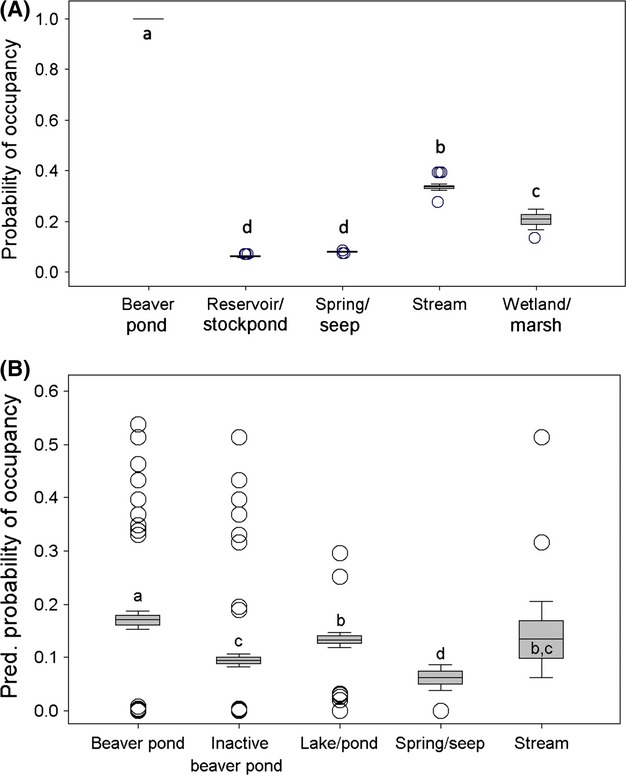
Average modeled probability of spotted frog occupancy by water body type based on (A) 78 water bodies in southwest Idaho and (B) 749 water bodies in Nevada. Boxes indicate ± 1 SE, bars indicate ± 2 SE, and open circles are water bodies ± 1 SD of mean. Probability estimates for each water body were derived from NPMR model predictions. For Idaho, sample sizes for each group are as follows (left to right): 5, 18, 13, 33, and 5 water bodies. Probability estimates could not be generated for 4 water bodies (2 backwaters/oxbows and 2 ponds), which occupied regions of predictor space with too few data to generate reliable model estimates. For Nevada, sample sizes for each group are as follows (left to right): 316, 222, 183, 9, and 19 water bodies. Groups with different letters are significantly different according to Duncan's multiple-range test.

In Idaho, occupancy was best predicted by an interaction between habitat type and maximum depth of water bodies (*P* = 0.02; Table3, Fig.3A). The probability of occupancy reached a maximum of 1.0 in beaver ponds and a minimum of 0.05 in shallow (MAXDEPTH < 1 m) reservoirs or stock ponds and shallow springs or seeps. Stream sites without beaver ponds had the next highest probability of occupancy (0.34), and the effect of maximum depth on occupancy was greatest in this habitat type, with shallow (0–0.5 m) and deep (2–2.5 m) stream reaches having occupancy probabilities of 0.28 and 0.40, respectively. Wetland or marsh habitats were half as likely to be occupied as streams (Fig.3A).

**Table 3:** Nonparametric multiplicative regression (NPMR) results for models predicting Columbia spotted frog occupancy in three states

State	*n* Sites	log*β*	Bootstrap results^1^	N^*^^2^	Predictor^3^	Sensitivity	Tolerance
ID	78	7.5	6.5 ± 0.22	15.9	(+) MAXDEPTH	0.07	1.5 (60%)
					(±) HABITAT	na	na
OR	75	9.9	9.7 ± 0.19	15.7	(±) HABITAT	na	na
					(^) VEGHT	0.1	24.7 (15%)
					(+) MAXDEPTH	0.05	0.7 (35%)
NV	778	16.6	18.8 ± 0.38	71.8	(+) MAXDEPTH	0.14	0.5 (20%)
					(s) EMERGVEG	0.08	10 (20%)
					(±) FISHSTATUS	na	na
					(±) LOTICLENTIC	na	na

1 Mean ± SE log*β* from 100 bootstrap resampling runs.

2 N^*^, the average neighborhood size, is the average number of sample units contributing to the estimate of occupancy at each point on the modeled surface.

3 Symbols in parentheses indicate the *general* direction of the relationship between each predictor and response variable: “+” indicates positive, “^” indicates Gaussian, “s” indicates sigmoidal, and “±” indicates that the variable is categorical (and that sensitivity and tolerance values are not applicable).

The best fitting model for Oregon sites was an interaction between habitat type, maximum depth, and emergent vegetation height (*P* = 0.02; Table3, Appendix 4). As in Idaho, beaver ponds were most likely to be occupied, followed by streams or rivers, ponds, reservoirs or stock ponds, and finally springs or seeps. Of the 57 sites with average emergent vegetation heights <27 cm, none were occupied by spotted frogs. Emergent vegetation heights at occupied sites averaged 47 cm (range = 27–82 cm), compared to an average of 19.7 cm (range = 0–165 cm) at unoccupied sites. Emergent vegetation height and percent cover were not correlated (*r* = 0.051).

**Figure 4:**
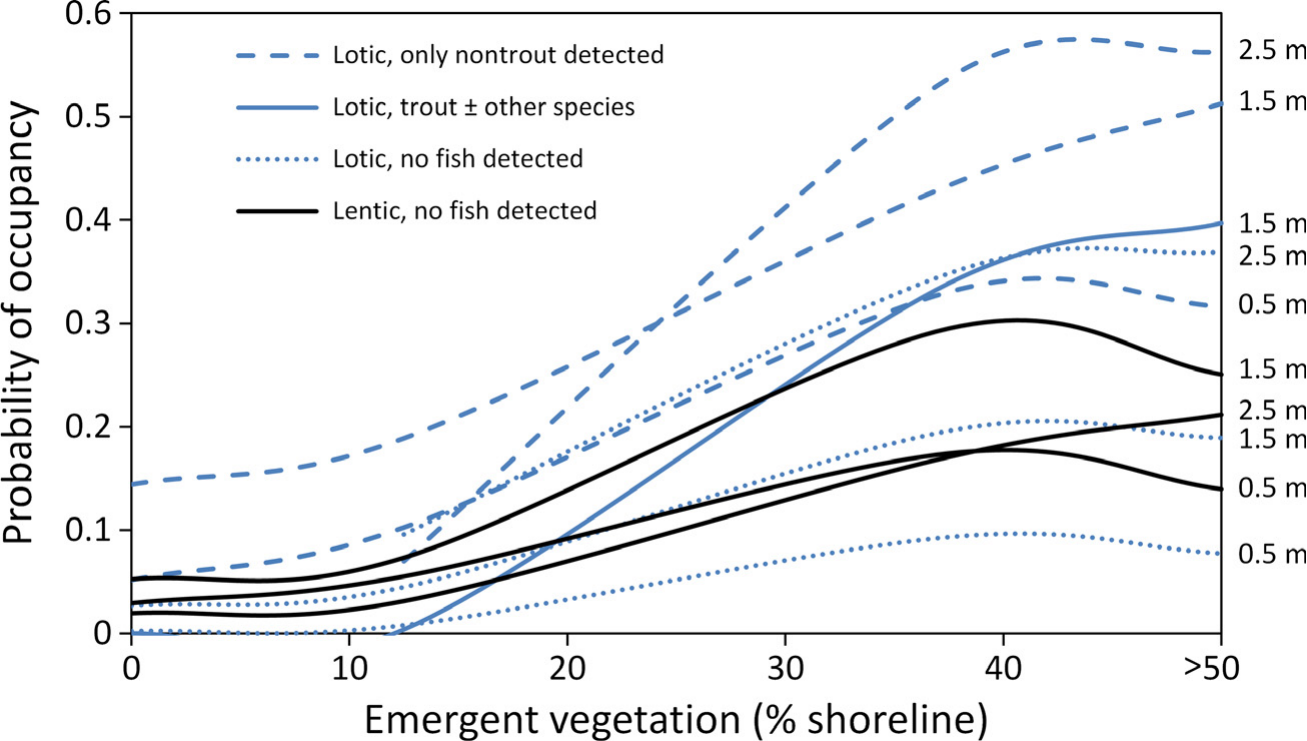
NPMR modeled relationship between probability of spotted frog occupancy and percent of shoreline containing emergent vegetation for sites in Nevada. Separate line series are given for each combination of lotic or lentic habitat (indicated by line color), fish occupancy status (indicated by line style), and maximum water body depth (indicated numerically at right of each series). Several combinations that were not observed or were observed too infrequently to produce reliable model estimates were omitted. Depths are categorical visual estimates from habitat surveys.

Nevada had a dataset 10 times larger than those of the other two states, which allowed for more predictor variables to be included. Occupancy was best predicted by maximum depth, percent of shoreline with emergent vegetation, the fish status of the water body, and whether the water body was lotic or lentic (*P* < 0.001; Table3, Fig.4). The highest probability of occupancy (0.56) was in deep (>2 m maximum depth), lotic water bodies that contained only nontrout fish species and had a high proportion of shoreline containing emergent vegetation (>50% shoreline coverage). Shallow (<0.5 m) sites with little emergent vegetation and trout present or no fish detected were the least likely to be occupied (Fig.4). Occupancy rates were relatively low in water bodies where emergent vegetation covered from 0 to 15% of shoreline and increased substantially as emergent vegetation increased, before leveling off. Lentic sites were far less likely to be occupied than most lotic sites, except when lotic sites were shallow (0.5–1.5 m maximum depth) and fishless. Maximum depth was particularly important in lotic water bodies where only nontrout fish were detected and in lotic water bodies where no fish were detected (Fig.4). Fish status had a strong influence on spotted frog occupancy as well, especially in sites with little emergent vegetation. At emergent vegetation values below 12.5%, the probability of occupancy was 0.0 in 1.5 m deep, lotic water bodies containing trout, compared to 0.20 in similar water bodies containing only nontrout fish (Fig.4). This positive nontrout fish effect diminished as emergent vegetation increased, but a 12% difference remained at the maximum value of emergent vegetation. Similar water bodies (i.e., lotic, 1.5 m maximum depth) where no fish were detected were more likely to be occupied than those with predatory trout when there was < 18% emergent vegetation, but as emergent vegetation increased, water bodies with trout were substantially (21%) more likely to be occupied than their fishless counterparts. To relate Nevada results to those of the other two states (where HABITAT was included as a predictor in final models), we performed a post hoc analysis and applied this model to habitat variables from each water body in Nevada (to generate an estimated probability of occupancy in each water body based on model results). We found that active beaver ponds had significantly higher predicted probability of occupancy than other habitat types, followed by lakes or ponds, and streams (Fig.3B).

### Threats to persistence

In all three states, beaver ponds had the highest naïve occupancy rate of all available habitat types. They constituted 57% of all occupied sites, and they were 11–66% more likely (depending on state) to be occupied than streams without beaver ponds (Appendix 2; see Table2 for a summary of results from this section).

Permanence of aquatic connectivity to a site (an inverse measure of habitat fragmentation for aquatic species) was positively related to occupancy of available sites. The most isolated sites (i.e., those classified as “isolated” or “temporary lentic,” depending on the dataset) were far less likely to be occupied (three-state average = 0.04) than the least isolated sites (three-state average = 0.37; Appendix 5). Further, 69% of all occupied sites were water bodies permanently connected to other sites via water.

Livestock grazing metrics were negatively related to spotted frog occupancy in general, with some differences among states (Appendix 6). Ungrazed sites in Oregon were 10.6 times more likely to be occupied than grazed sites, and 71% of occupied sites in Oregon were ungrazed. Cattle use (measured by feces cover at shoreline) was negatively correlated with emergent vegetation height (but not cover) and with spotted frog occupancy (*n* = 66, log*β* = 1.1, *P* = 0.02; Appendix 7). In Idaho, sites with “heavy” structural or water quality grazing impacts were just as likely to be occupied as sites with “light” impacts; however, 77% of all occupied sites had grazing impacts rated as “none” or “light.” In Nevada, nearly 50% of all occupied sites had “low” grazing impacts and 86% of occupied sites were rated either “low” or “moderate.”

Sites where trout were detected were 10–17% (depending on state) less likely to be occupied by spotted frogs than sites where only nontrout fish species were detected (Appendix 8). In Idaho and Nevada, sites with only nontrout fish accounted for a greater (over two times) proportion of occupied sites than did sites containing trout. Based on occurrence data from GIS databases (i.e., not from our habitat surveys), native redband and cutthroat trout tended to occupy areas with greater shoreline density, cooler annual air temperatures, and greater annual precipitation than was observed for 145 spotted frog breeding sites, but with substantial overlap with spotted frogs along these gradients (Fig.2, Appendix 9). There was very little overlap between spotted frog breeding locations and the two trout species at the water body level, as only 11% of breeding sites fell within 50 m of trout-occupied stream segments.

Bullfrogs were detected at seven sites surveyed for spotted frogs in Nevada, one of which (a beaver pond) was occupied by spotted frogs. This occupancy rate (0.14) is less than half the spotted frog occupancy rate (0.32) observed in 91 sites where a native frog species (*Pseudacris regilla*) was detected (*P. regilla* does not prey on amphibians). Based on occurrence data from GIS databases, bullfrogs tended to occupy slightly warmer, drier areas with less shoreline habitat than was observed for spotted frog breeding sites, but we found substantial overlap between the two species along these gradients (Fig.2, Appendix 9). Forty-one percent of spotted frog breeding sites were within 20 km of a bullfrog observation, and 87% fell within 80 km (Appendices 10a and 11). Of eight locations in Nevada with historic GIS records of spotted frogs both upstream and downstream of a more recent bullfrog observation, 7 (88%) have not had a subsequent spotted frog detection downstream of the bullfrog observation (average = 2.9 downstream, post-bullfrog surveys), whereas spotted frogs still occur upstream at each site.

Bd has been detected in amphibians at 47% of the 32 sites in the study area that have been tested and submitted to the Bd-maps database (Appendix 10B). Spotted frogs and bullfrogs have tested positive for Bd at 6 and 5 sites, respectively. Forty-six percent of spotted frog breeding sites are within 20 km of a Bd detection, and 87% are within 80 km (Appendix 11).

Droughts are expected to have a negative impact on spotted frog occupancy (Appendix 12), as sites that were classified as temporary (i.e., likely to dry in most or all years) had a 0.09 overall occupancy rate, whereas the probability of occupancy in permanently wet sites was 0.14 overall (but 0.33 in Idaho and 0.23 in Oregon). Spotted frogs were strongly associated with permanent hydroperiods, which accounted for 83% of all occupied sites.

## Discussion

### Habitat availability

A small portion of the Great Basin study area (5.4%) has both suitable climate and at least some perennial water needed for Columbia spotted frog occupancy, and many of these suitable habitats are distributed in relatively isolated patches across this vast, arid landscape. We found that the majority of the area of climate suitable for spotted frogs occurs on BLM land, which tended to be relatively water-poor. Perennial water tended to be more abundant on private, state, and USFS land. As early Euro-American settlement of the region occurred disproportionately near permanent water sources, private lands (which were not surveyed extensively for our datasets) could be particularly important for persistence of the spotted frog in the Great Basin. Private lands are recognized as critically important for the conservation of biodiversity globally because public lands are often not large enough (e.g., some parks and reserves) or do not provide sufficient habitat or landscape connectivity required for species persistence (Kamal et al. 2015). Assuming suitable habitat is available, higher elevation USFS land may become increasingly important as the region's climate becomes warmer and more arid, as is expected under most climate projections (Cayan et al. 2010; Cook et al. 2015). BLM land, although relatively water-poor, contains many sites currently occupied by spotted frogs and may play an important role in facilitating current and future connectivity. Our findings on connectivity (discussed below) underscore the importance of permanent aquatic connectivity for site-level occupancy. Further, recent findings from Pilliod et al. (in press) indicate that connectivity and gene flow are already low and problematic in this region at the edge of the species’ distribution.

Our analyses also suggest that many large, suitable patches within the study area are not currently occupied and could be used as reintroduction sites if necessary. The reasons for a lack of occupancy in these patches are uncertain, but our analyses suggest that these patches are sufficiently large, have sufficient perennial water, and have suitable climate conditions to support spotted frog occupancy. Amphibian reintroductions elsewhere have had reasonable successes, and practices are improving with implementation of adaptive management principles (Ewen et al. 2014).

### Water body-scale habitat associations

Spotted frog occupancy at the water body scale was predicted by similar habitat variables in all three states, suggesting similar drivers of occupancy across the region. Lotic habitats, especially beaver ponds with deep maximum depth, abundant shoreline vegetation, and nontrout fish species, are particularly important habitats for Columbia spotted frogs in the Great Basin.

The relative importance of lotic habitat (or more precisely, lentic areas within lotic habitats) is somewhat unique to the Great Basin population, as studies have found Columbia spotted frogs in the Northern clade to be more strongly associated with pond and lake habitats (Pilliod et al. 2010a). The high value of Great Basin stream habitats relative to lentic sites could be due to longer hydroperiods and increased connectivity in this comparatively arid region of the species’ range. For example, 77% of Idaho stream sites surveyed had permanent aquatic connectivity, compared to only 28% of lentic sites, and in Nevada, 86% of stream sites had permanent hydroperiods, compared to 56% of lentic sites. This is consistent with other studies in the region that found spotted frogs to be associated with oxbows or other sites with permanent hydroperiods (Munger et al. 1998; Hossack et al. 2013). Further supporting the importance of permanent water was our observation that streams containing fish (an indication of either water permanence or at least temporary connectivity to permanent water) were at least twice as likely to be occupied by spotted frogs as fishless sites of equal depth and vegetation characteristics. However, permanent water bodies often also contain predators, like trout and bullfrogs, which may reduce recruitment and population growth rates in spotted frog populations compared with water bodies with semi-permanent hydroperiods (McCaffery et al. 2014; Ryan et al. 2014). Hence, landscapes that provide a range of water body hydroperiods may increase population resistance to effects of predators and resilience to risks associated with prolonged drought (McCaffery et al. 2014).

Greater maximum depth of water bodies is associated with greater hydroperiod permanency, increased aquatic escape cover from predators, and overwintering habitat beneath ice (Bull and Hayes 2002). Either a lack of permanent water or overwintering habitat can preclude spotted frog occupancy of fairly large areas (e.g., catchments) of otherwise suitable habitat, even when they use a complementary-resource approach to season-specific habitat (Pilliod et al. 2002). We found deep (e.g., >2 m maximum depth) water bodies to be relatively scarce in the Great Basin, where many streams have been diverted for irrigation or channelized and downcut through various riparian management practices (Chambers and Miller 2004). Maximum depth was an important predictor of occupancy in all three states, and increasing the availability of deep water bodies could be a primary means of increasing spotted frog resistance and resilience to seasonal drought and longer-term aridification of the region (Cayan et al. 2010).

The importance of emergent vegetation as foraging areas with low predator efficiency, cover for hiding and thermoregulation, and as sites for egg mass deposition and larval rearing has been noted in several studies (Licht 1971, 1975; Pilliod et al. 2002, 2010a; Welch and MacMahon 2005; Pearl et al. 2007). We found evidence that emergent vegetation offsets negative effects of predatory trout, as the difference in spotted frog occupancy rates between sites with and without trout diminished with increasing emergent vegetation coverage in otherwise similar sites. Studies of other amphibians have found negative effects of salmonids (Pearl et al. 2009a; Pilliod et al. 2012) and that emergent vegetation becomes increasingly important to amphibians such as long toed salamanders (*Ambystoma macrodactylum*) when predatory fish are present in the catchment (Pilliod et al. 2013). The benefit of emergent vegetation cover observed here diminished after approximately 50% of the shoreline contained emergent vegetation (i.e., Nevada sites), or after about 20% of the site surface contained emergent vegetation (i.e., Idaho and Oregon sites). Our findings also indicate that emergent vegetation height is important, as decreased height (which was correlated with increased grazing pressure) was associated with low spotted frog occupancy rates. Other studies have found similar effects of grazing on emergent vegetation height, but they found no corresponding effect on spotted frog reproduction or survival parameters (Bull and Hayes 2000; Adams et al. 2009), possibly because the effects of livestock grazing are dependent on site characteristics, grazing intensity, and landscape context. We suspect that emergent vegetation height may be important for cover from aerial predators or for thermoregulation.

Finally, certain aquatic habitat types tend to have optimal combinations of habitat elements more frequently than others. Beaver ponds, in particular, were significantly more likely to be occupied than any other habitat type in all three states, including stream sites without beaver ponds and stream sites with inactive beaver ponds. This suggests that manipulation of stream habitats by beaver (and not simply streams in general or streams selected by beaver) is primarily responsible for increased spotted frog habitat quality. Ecosystem engineering by beaver may affect spotted frog habitat at multiple scales (Appendix 13). First, by manipulating site-scale habitat elements (e.g., increasing water body depth, permanence, shoreline area, and emergent vegetation), beaver increase the quality of individual sites for spotted frogs (Hossack *et al*. 2015). Second, by constructing chains of ponds along stream networks, beaver increase the quantity, hydroperiod, and connectivity of sites within and across catchments (Gibson and Olden 2014). This notion is supported by a study that found positive effects of beaver pond habitat on amphibians at both the site and landscape scale in boreal streams (Stevens et al. 2007). Site- and landscape-level habitat changes generated by beaver may increase the resilience of Great Basin spotted frog populations to potential threats, including drought, climate changes, intensive livestock grazing, and predators (both native and non-native). Beaver reintroductions have benefitted multiple amphibian species elsewhere (e.g., Dalbeck et al. 2007) in addition to several, localized spotted frog populations in the Great Basin (Lingo 2013, Hossack et al. 2013).

### Threats to persistence

Based on our findings and those of similar studies, loss of beaver habitat (Stevens et al. 2007; Gibson and Olden 2014; Hossack et al. 2015), loss of aquatic linkages (Funk et al. 2008; Pilliod et al. 2010a, in press) high-impact livestock grazing, salmonids (Pearl et al. 2009a; Pilliod et al. 2013), and drought (Hossack et al. 2013) are expected to have fairly strong negative impacts on spotted frog occupancy. The threats examined here may also interact cumulatively to influence spotted frog persistence within the Great Basin, especially if climate changes and human resource uses add additional stress to populations (Ryan et al. 2014).

Within areas of available habitat, we found considerable overlap between spotted frogs, native salmonid predators, and non-native bullfrogs. This combination of predators has the potential to act like a vice, with predatory trout potentially excluding spotted frogs from the upper elevations (Pilliod et al. 2010a) and predatory bullfrogs (and probably non-native warm-water fishes such as bass) excluding spotted frogs from lower elevations (Pearl et al. 2004). Competition or predation by these species may be excluding spotted frogs from otherwise suitable habitat, limiting interpopulation movements, and further reducing population sizes at the margins of their Great Basin distribution.

We also found evidence that most spotted frog breeding sites are in relatively close proximity to known native trout (nearly 70% of spotted frog breeding catchments [HUC-12] contain trout), bullfrog, or Bd pathogenic fungus locations. Although there is inadequate survey information from which to draw strong conclusions about the magnitude of bullfrog and Bd threats to spotted frogs in the Great Basin, the effectiveness of bullfrogs as both competitors and predators of spotted frog species and their capacity to carry Bd are well documented (Pearl et al. 2004, 2009b; Garner et al. 2006; Monello et al. 2006). Further, bullfrogs, Bd, and trout appear to be capable of occupying the range of temperature and precipitation conditions that are currently occupied by spotted frogs. Preventing bullfrogs (and the Bd they often carry) from dispersing and coming into equilibrium with their potential climatic niche (through colonizing additional spotted frog habitat, particularly at higher elevations) could clearly benefit spotted frog populations in the region. Bullfrogs can be adept dispersers. For example, in a Montana (USA) floodplain, they expanded their range by up to 39 km over a 3-year period (Sepulveda et al. 2014).

We suspect that several of the threats evaluated could be marginalized or mitigated through the increased habitat quality, quantity, and connectivity that could come with widespread beaver reintroductions (Dalbeck et al. 2007; Gibson and Olden 2014; Pollock et al. 2015) and careful wetland and riparian management and restoration (Hossack et al. 2013). Potential impacts from salmonids, bullfrogs, and chytridiomycosis may continue into the foreseeable future regardless of (or possibly increase because of) large-scale beaver reintroduction, but the habitat changes created by beaver could also greatly increase spotted frog resistance and resilience to these biotic threats, primarily by boosting the number, sizes, and distribution of populations.

## Conclusions

Persistence on the southern end of this species’ 2,700-km latitudinal distribution is largely facilitated by habitat stability (i.e., permanent hydroperiod) and connectivity, predator-free refugia (or escape cover in areas containing predators), and an ecosystem engineer (i.e., beaver) that modifies habitat quantity, quality, and connectivity. These factors likely combine to increase Columbia spotted frog resistance and resilience to habitat and climate changes, biotic invasions, and stochastic or anthropogenic disturbances, effectively buffering Great Basin populations from distributional “erosion” in the southernmost portion of the species’ range. Understanding factors that mitigate the interactive effects of harsh climatic conditions and anthropogenic disturbances could improve conservation efforts aimed at preserving species or populations persisting in marginal, but geographically important locations.

## Figures and Tables

**Figure:**
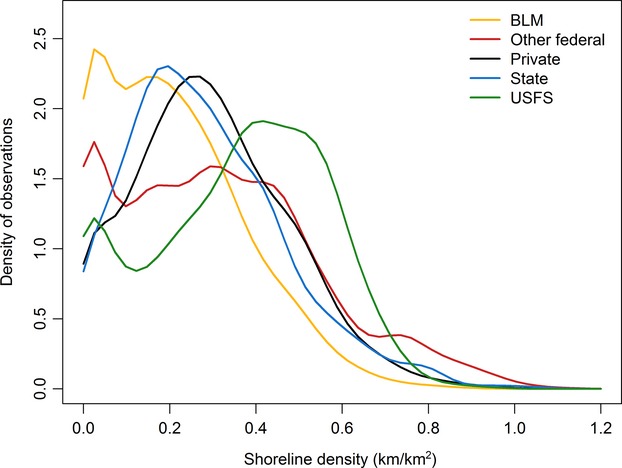
Kernel smoothed probability density of perennial shoreline density values, by land owner, within areas of the Great Basin having suitable climate for spotted frog breeding. Shoreline density was calculated using a 5-km moving window around each 270-m pixel. Abbreviations are as follows: BLM: Bureau of Land Management; and USFS: U.S. Forest Service.

**Figure:**
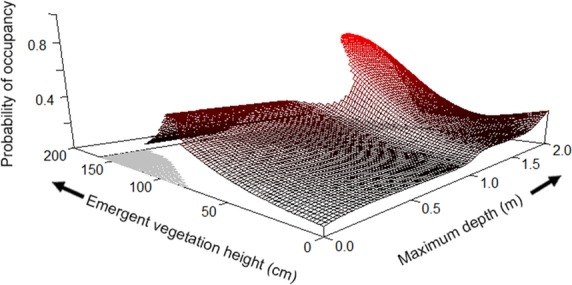
NPMR modeled relationship between probability of spotted frog occupancy (vertical axis) and average emergent vegetation height and maximum depth. Model based on 75 water bodies in Oregon. Gray areas indicate regions of predictor space with too few data points for reliable estimates to be made.

**Figure:**
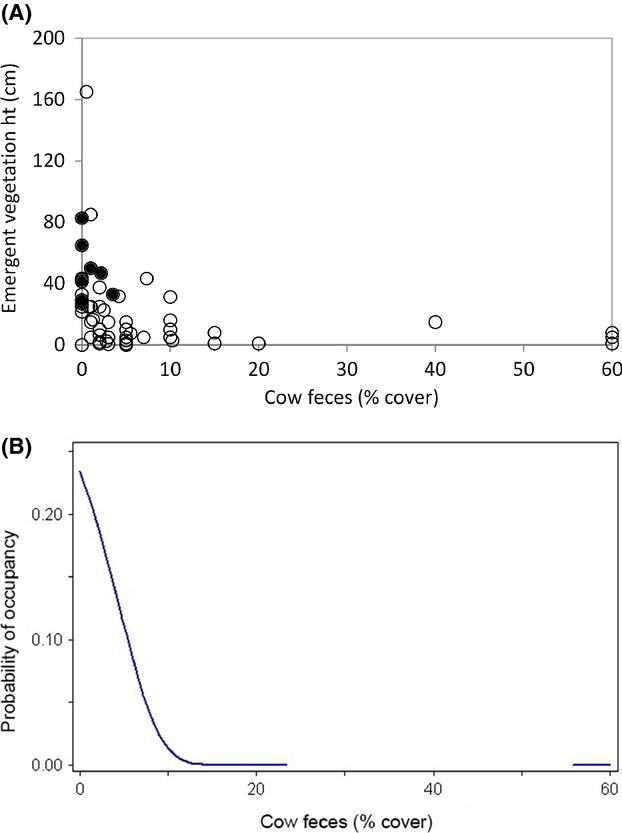
Emergent vegetation height and cow feces cover measured in paired 2-m quadrats at 66 sites in Oregon. (A) Average height of emergent vegetation and average percent cover of cow feces. Solid points are sites occupied by spotted frogs. Within-site error bars omitted for clarity. (B) NPMR modeled probability of spotted frog occupancy and cow feces cover along shoreline. Fitted line is discontinuous in regions of predictor space with too few data points for reliable estimates to be made. For each pair, one quadrat was placed along the shoreline and the second quadrat was placed immediately adjacent to the first, but inside the water. The number of replicate quadrat pairs within sites varied from 1 to 11 (average = 2.5 replicates per site), depending on the size of the site.

**Figure:**
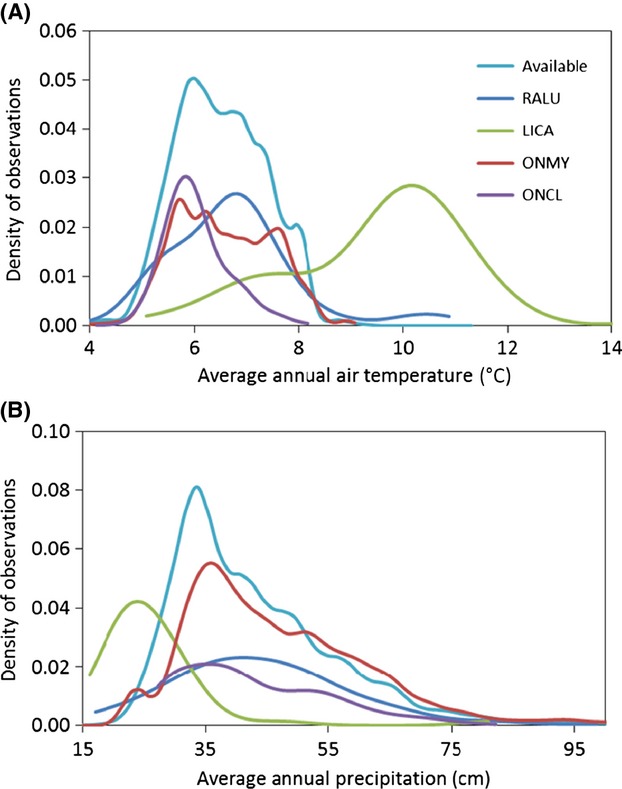
Kernel smoothed probability density of (A) 30-year average temperature values (1981–2010) and (B) 30-year average temperature values (1981–2010), within areas of the Great Basin having suitable climate for spotted frog breeding. “Available” refers to all pixels within suitable spotted frog breeding habitat. Sample sizes for distribution calculations were as follows: Available = 412,634 pixels; RALU = 145 observed breeding sites; LICA = 182 observed occurrence points; ONMY = 111,561 occupied stream vertices; and ONCL = 11,428 occupied stream vertices.

**Figure:**
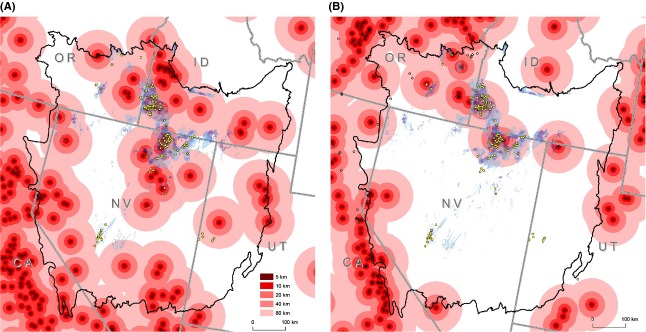
Distribution of (A) American bullfrog (*Lithobates catesbeianus*) observations and (B) amphibian chytrid fungus detections in and around the Great Basin, buffered by varying distances (shades of red). Also shown are spotted frog breeding sites (yellow points) and perennial shoreline density (gray-blue color ramp; see Fig.1 for details). Data sources are provided in Table1. Spotted frog breeding sites in Utah are not a part of the Great Basin Distinct Population Segment, but are shown because of their proximity.

**Figure:**
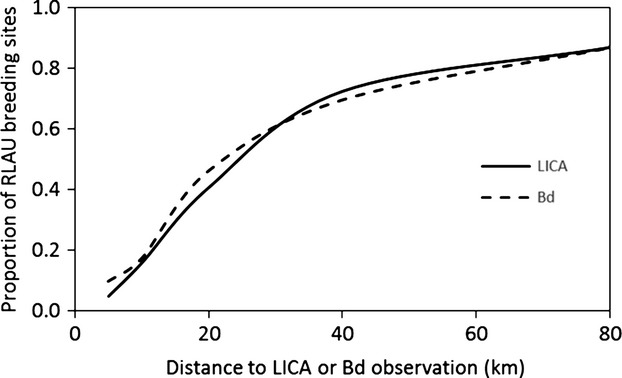
Proportion of 145 spotted frog breeding sites vs. distance from American bullfrog (LICA) or amphibian chytrid fungus (Bd) detections in and around the Great Basin. LICA and Bd data sources are provided in Table1. Utah sits were not included in calculations.

**Figure:**
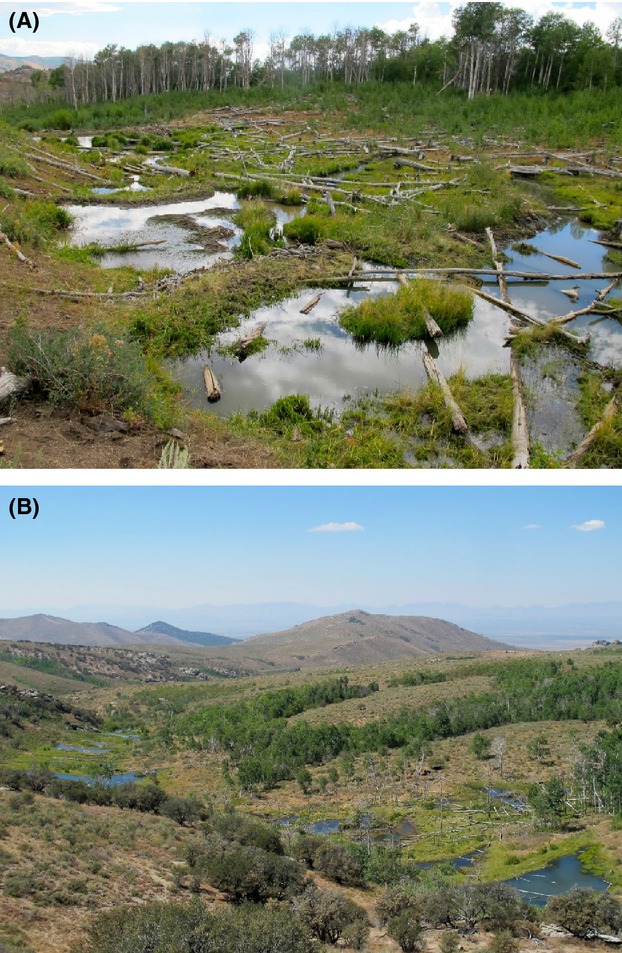
(A) Manipulation of stream habitats by beaver affects spotted frog habitat quality at the site scale, (B) while chains of beaver dams on single and neighboring streams increase aquatic habitat quantity, connectivity, and hydroperiod at the landscape scale. Photos by Brock Dolman, used with permission.

## Appendix 1

**Table:** For each dataset (i.e., State), habitat variables collected at each water body and included as potential predictor variables in NPMR analyses of spotted frog occupancy. Variables are grouped into several functional categories for clarity.

Variable category	Variable	Units	Description
ID			
Species detected	RALUOCC	Binary	Observed occupancy of Columbia spotted frogs (RALU), where 0 = not detected and 1 = detected
	FISHOCC	Binary	Observed occupancy of the site by any species of fish, where 0 = not detected and 1 = detected
	FISHSTATUS	Categorical	Whether the site was occupied by trout (±other species), only nontrout fish, or no fish were detected
	PREDOCC	Binary	Observed occupancy of the site by any species known to prey on RALU (e.g., garter snakes, bullfrogs)
	GRAZEIMPACT	Categorical	Whether grazing impacts to the site were none, light, heavy structural, or heavy structural and water quality
Habitat structure	HABITAT	Categorical	Whether the site was an active/inactive beaver pond, backwater/oxbow, pond, reservoir/stock pond, spring/seep, stream, or wetland/marsh habitat
	LOTICLENTIC	Categorical	Whether the site was part of a lotic (stream, backwater/oxbow, beaver pond) or a lentic system (all other HABITAT types)
	MAXDEPTH	Meters	Maximum depth of the site
	G2M	Percent	Percent of the site greater than 2 m deep
	L50CM	Percent	Percent of the site less than 50 cm deep
	SUBSTRATE	Categorical	The dominant substrate of the site (silt/mud, sand, gravel, cobble, boulder/bedrock)
	ALGAE	Percent	Percent of the water surface covered by algae
	EMERGVEG	Percent	Percent of the *water surface* containing emergent vegetation
	WILLOW	Percent	Percent of the shoreline containing willows
	SITEORI	Categorical	Site origin (natural or man-made)
Connectivity/hydroperiod	SITEDRY	Categorical	Whether site was dry at time of survey
	WATERCON	Categorical	Whether the site was isolated, temporarily connected, or permanently connected to other sites via water
	HYDROPERIOD	Categorical	Temporarily or permanently wet
Water chemistry	ALKALINITY	ppm	Water alkalinity at the time of the survey
	HARDNESS	ppm	Water hardness at the time of the survey
	NITRATE	ppm	Water nitrate concentration at the time of the survey
	NITRITE	ppm	Water nitrite concentration at the time of the survey
	PH	-	Water pH at the time of the survey
OR			
Species detected	RALUOCC	Binary	Observed occupancy of Columbia spotted frogs, where 0 = not detected and 1 = detected
	FISHOCC	Binary	Observed occupancy of the site by any species of fish, where 0 = not detected and 1 = detected
	FISHSTATUS	Categorical	Whether the site was occupied by trout (±other species), only nontrout fish, or no fish were detected
	GRAZED	Binary	Whether the site had evidence (e.g., hoof prints, feces) of cattle grazing
Habitat structure	HABITAT	Categorical	Whether the site was a beaver pond, pond, reservoir/stock pond, spring/seep, or stream/river habitat
	LOTICLENTIC	Categorical	Whether the site was part of a lotic (stream, backwater/oxbow, beaver pond) or a lentic system (all other HABITAT types)
	MAXDEPTH	Meters	Maximum depth of the site
	G2M	Percent	Percent of the site greater than 2 m deep
	L50CM	Percent	Percent of the site less than 50 cm deep
	AREA	m^2^	Approximate wetted surface area of the site, derived from site length and width measurements
	EMERGVEG	Percent	Percent of the *water surface* containing emergent vegetation
	QUADVEGCOV	Percent	Average percent cover of emergent vegetation within 2-m quadrats placed in the water along the shoreline. Number of quadrats per site ranged 1–11 depending on site size (average = 2.5 replicate quadrats per site)
	QUADVEGHT	cm	Average height of emergent vegetation within 2-m quadrats placed *in the water* along the shoreline. Number of quadrats per site ranged 1–11 depending on site size (average = 2.5 replicate quadrats per site)
	QUADCOWCOV	Percent	Average percent cover of cattle feces within 2-m quadrats placed *outside of the water* along the shoreline. Number of quadrats per site ranged 1–11 depending on site size (average = 2.5 replicate quadrats per site)
	FREQBOULDER	Frequency	Frequency (%) of quadrats inside the water, with boulders as the dominant substrate
	FREQCOBBLE	Frequency	Frequency (%) of quadrats inside the water, with cobble as the dominant substrate
	FREQGRAVEL	Frequency	Frequency (%) of quadrats inside the water, with gravel as the dominant substrate
	FREQLFGR	Frequency	Frequency (%) of quadrats inside the water, with leaves or grass as the dominant substrate
	FREQMUD	Frequency	Frequency (%) of quadrats inside the water, with mud as the dominant substrate
	FREQSAND	Frequency	Frequency (%) of quadrats inside the water, with sand as the dominant substrate
	FREQWOOD	Frequency	Frequency (%) of quadrats inside the water, with down woody debris as the dominant substrate
	SITEORI	Categorical	Whether site was man-made
Connectivity/hydroperiod	HYDROPERIOD	Categorical	Temporarily or permanently wet
Water chemistry	PH	–	Water pH at the time of the survey
	CONDUCTIVITY	uS	Water conductivity at the time of the survey
	TURBID	Categorical	Whether water was turbid at the time of the survey
NV			
Species detected	RALUOCC	Binary	Observed occupancy of Columbia spotted frogs, where 0 = not detected and 1 = detected
	FISHOCC	Binary	Observed occupancy of the site by any species of fish, where 0 = not detected and 1 = detected
	FISHSTATUS	Categorical	Whether the site was occupied by trout (±other species), only nontrout fish, or no fish were detected
	GRAZEIMPACT	Categorical	Only for sites occupied by RALU, whether cattle grazing impacts to vegetation and water quality were low, moderate, or high
Habitat structure	HABITAT	Categorical	Whether the site was an active beaver pond, inactive beaver pond, lake/pond, stream, or wetland/marsh habitat
	MAXDEPTH	Meters	Maximum depth of the site
	SUBSTRATE	Categorical	The dominant substrate of the site (silt/mud, sand, gravel, cobble, boulder/bedrock)
	EMERGVEG	Percent	Percent of the *shoreline* containing emergent vegetation
	NSHALLOWS	Categorical	Whether the northern shoreline was shallow (less than 50 cm deep)
	NVEG	Categorical	Whether the northern shoreline contained emergent vegetation
	SITEORI	Categorical	Whether site was natural or man-made
Connectivity/hydroperiod	HYDROPERIOD	Categorical	Temporarily or permanently wet based on field observations and wetland inventory data

## Appendix 2

**Table:** Spotted frog occupancy rates by habitat type and state.

State	Habitat type	*n* Sites	*n* Occupied sites	Naïve occupancy rate	Proportion of occupied sites
ID	Active/inactive beaver pond	5	5	1.00	0.23
	Backwater/oxbow	2	2	1.00	0.09
	Pond	2	1	0.50	0.05
	Res/stockpond	18	1	0.06	0.05
	Spring/seep	13	1	0.08	0.05
	Stream	33	11	0.33	0.50
	Wetland/marsh	5	1	0.20	0.05
NV	Beaver pond (active)	406	70	0.17	0.54
	Beaver pond (inactive)	236	15	0.06	0.12
	Lake/pond	288	29	0.10	0.22
	Stream	116	7	0.06	0.05
	Wetland/marsh	8	1	0.13	0.01
OR	Beaver pond	6	5	0.83	0.36
	Pond	9	2	0.22	0.14
	Res/stockpond	61	1	0.02	0.07
	Spring/seep	2	0	0.00	0.00
	Stream/river	18	6	0.33	0.43

## Appendix 5

**Table:** Spotted frog occupancy rates by fragmentation/connectivity class and state.

State	Fragmentation class	*n* Sites	*n* Occupied sites	Naïve occupancy rate	Proportion of occupied sites
ID	Isolated	23	0	0.00	0.00
	Temp connected	14	5	0.36	0.23
	Perm connected	41	17	0.41	0.77
NV	Temp lentic	131	16	0.12	0.13
	Temp lotic	105	9	0.09	0.07
	Perm lentic	167	17	0.10	0.14
	Perm lotic	642	81	0.13	0.66
OR	Temp lentic	26	0	0.00	0.00
	Temp lotic	5	0	0.00	0.00
	Perm lentic	43	3	0.07	0.21
	Perm lotic	19	11	0.58	0.79

## Appendix 6

**Table:** Spotted frog occupancy rates by livestock grazing impact class and state.

State	Grazing impact class	*n* Sites	*n* Occupied sites	Naïve occupancy rate	Proportion of occupied sites
ID	None	26	5	0.19	0.23
	Light	33	12	0.36	0.55
	Heavy structural	7	2	0.29	0.09
	Heavy structural & water quality	8	3	0.38	0.14
	Unknown	4	0	0	0
NV	Low	–	44	–	0.49
	Moderate	–	33	–	0.37
	High	–	13	–	0.14
OR	Ungrazed	19	10	0.53	0.71
	Grazed	77	4	0.05	0.29

## Appendix 8

**Table:** Spotted frog occupancy rates by fish status^1^ class and state.

State	Fish status	*n* Sites	*n* Occupied sites	Naïve occupancy rate	Proportion of occupied sites
ID	No fish detected	48	10	0.21	0.45
	Trout detected ± other fish	9	3	0.33	0.14
	Only nontrout detected	21	9	0.43	0.41
NV	No fish detected	936	82	0.09	0.63
	Trout detected ± other fish	62	9	0.15	0.07
	Only nontrout detected	119	36	0.30	0.27
OR	No fish detected	79	5	0.06	0.36
	Trout detected ± other fish	14	7	0.50	0.50
	Only nontrout detected	3	2	0.67	0.14

Fish species detected during surveys were rainbow trout (*Oncorhynchus mykiss*), redband trout (*O. mykiss* spp. *gairdneri*), Lahontan cutthroat trout (*O. clarkii* spp. *henshawi*), brook trout (*Salvelinus fontinalis*), speckled dace (*Rhinichthys osculus*), sculpin (*Cottus* sp.), and sucker (*Catostomus* sp.).

## Appendix 12

**Table:** Spotted frog occupancy rates by hydroperiod class and state.

State	Hydroperiod	*n* Sites	*n* Occupied sites	Naïve occupancy rate	Proportion of occupied sites
ID	Temporary	21	3	0.14	0.14
	Permanent	57	19	0.33	0.86
NV	Temporary	243	25	0.10	0.20
	Permanent	813	102	0.13	0.80
OR	Temporary	31	0	0.00	0.00
	Permanent	62	14	0.23	1.00

## References

[b1] Adams MJ, Pearl CA, McCreary B, Galvan SK, Wessell SJ, Wente WH (2009). Short-term effect of cattle enclosures on Columbia spotted frog (*Rana luteiventris*) populations and habitat in northeastern Oregon. J. Herpetol.

[b2] Adams MJ, Chelgren ND, Reinitz D, Cole RA, Rachowicz LJ, Galvan S (2010). Using occupancy models to understand the distribution of an amphibian pathogen, *Batrachochytrium dendrobatidis*. Ecol. Appl.

[b3] Arkle RS, Pilliod DS, Hanser SE, Brooks ML, Chambers JC, Grace JB (2014). Quantifying restoration effectiveness using multi-scale habitat models: implications for sage-grouse in the Great Basin. Ecosphere.

[b4] Bos DH, Sites JW (2001). Phylogeography and conservation genetics of the Columbia spotted frog (*Rana luteiventris*; Amphibia, Ranidae). Mol. Ecol.

[b5] Bull EL, Hayes MP (2000). Livestock effects on reproduction of the Columbia spotted frog. J. Range Manag.

[b6] Bull EL, Hayes MP (2002). Overwintering of Columbia spotted frogs in Northeastern Oregon. Northwest Sci.

[b7] Cayan DR, Das T, Pierce DW, Barnett TP, Tyree M, Gershunov A (2010). Future dryness in the southwest US and the hydrology of the early 21st century drought. Proc. Natl Acad. Sci. USA.

[b8] Chambers JC, Miller JR (2004). Great Basin riparian ecosystems: ecology, management, and restoration.

[b9] Clements C (1991). Beavers and riparian ecosystems. Rangelands.

[b10] Cook BI, Ault TR, Smerdon JE (2015). Unprecedented 21st century drought risk in the American Southwest and Central Plains. Sci. Adv.

[b11] Cunningham JM, Calhoun AJ, Glanz WE (2007). Pond-breeding amphibian species richness and habitat selection in a beaver-modified landscape. J. Wildl. Manag.

[b12] Dalbeck L, Luscher B, Ohlhoff D (2007). Beaver ponds as habitat of amphibian communities in a central European highland. Amphibia-Reptilia.

[b13] Ewen J, Soorae P, Canessa S (2014). Reintroduction objectives, decisions and outcomes: global perspectives from the herpetofauna. Anim. Conserv.

[b14] Fenton NJ, Bergeron Y (2008). Does time or habitat make old-growth forests species rich? Bryophyte richness in boreal *Picea mariana* forests. Biol. Conserv.

[b15] Funk WC, Pearl CA, Draheim HM, Adams MJ, Mullins TD, Haig SM (2008). Range-wide phylogeographic analysis of the spotted frog complex (*Rana luteiventris* and *Rana pretiosa*) in northwestern North America. Mol. Phylogenet. Evol.

[b16] Garner TW, Perkins MW, Govindarajulu P, Seglie D, Walker S, Cunningham AA (2006). The emerging amphibian pathogen *Batrachochytrium dendrobatidis* globally infects introduced populations of the North American bullfrog, *Rana catesbeiana*. Biol. Lett.

[b17] Gerick AA, Munshaw RG, Palen WJ, Combes SA, O'Regan SM (2014). Thermal physiology and species distribution models reveal climate vulnerability of temperate amphibians. J. Biogeogr.

[b18] Gibson PP, Olden JD (2014). Ecology, management, and conservation implications of North American beaver (*Castor canadensis*) in dryland streams. Aquat. Conserv.

[b19] Grundel R, Pavlovic NB (2007). Response of bird species densities to habitat structure and fire history along a midwestern open-forest gradient. Condor.

[b20] Hossack BR, Adams MJ, Pearl CA, Wilson KW, Bull EL, Lohr K (2013). Roles of patch characteristics, drought frequency, and restoration in long-term trends of a widespread amphibian. Conserv. Biol.

[b21] Hossack BR, Gould WR, Patla DA, Muths E, Daley R, Legg K (2015). Trends in Rocky Mountain amphibians and the role of beaver as a keystone species. Biol. Conserv.

[b22] Hutchinson GE (1957). Concluding remarks. Cold Spring Harbor Symp. Quant. Biol.

[b23] Kamal S, Grodzińska-Jurczak M, Brown G (2015). Conservation on private land: a review of global strategies with a proposed classification system. J. Environ. Plan. Manag.

[b24] Karraker NE, Gibbs JP (2009). Amphibian production in forested landscapes in relation to wetland hydroperiod: a case study of vernal pools and beaver ponds. Biol. Conserv.

[b25] Licht LE (1971). Breeding habits and embryonic thermal requirements of the frogs, *Rana aurora aurora* and *Rana pretiosa pretiosa*, in the pacific northwest. Ecology.

[b26] Licht LE (1975). Comparative life history features of the western spotted frog, *Rana pretiosa*, from low-and high-elevation populations. Can. J. Zool.

[b100] Lingo HA (2013). http://scholarworks.boisestate.edu/td/870.

[b27] May BE, Writer BJ, Albeke S (2012). Redband trout status update summary.

[b28] McCaffery RM, Eby LA, Maxell BA, Corn PS (2014). Breeding site heterogeneity reduces variability in frog recruitment and population dynamics. Biol. Conserv.

[b29] McCune B (2006). Non-parametric habitat models with automatic interactions. J. Veg. Sci.

[b30] McCune B (2009). http://www.pcord.com/NPMRintro.pdf.

[b31] McCune B, Mefford MJ (2009). HyperNiche. Multiplicative habitat modeling. Version 2.22.

[b32] Monello RJ, Dennehy JJ, Murray DL, Wirsing AJ (2006). Growth and behavioral responses of tadpoles of two native frogs to an exotic competitor, *Rana catesbeiana*. J. Herpetol.

[b33] Munger JC, Gerber M, Madrid K, Carroll MA, Petersen W, Heberger L (1998). US national wetland inventory classifications as predictors of the occurrence of Columbia spotted frogs (*Rana luteiventris*) and Pacific treefrogs (Hylaregilla). Conserv. Biol.

[b34] Olson DH, Aanensen DM, Ronnenberg KL, Powell CI, Walker SF, Bielby J (2013). Mapping the global emergence of *Batrachochytrium dendrobatidis*, the amphibian chytrid fungus. PLoS ONE.

[b35] Pearl CA, Adams MJ, Bury RB, McCreary B (2004). Asymmetrical effects of introduced bullfrogs (*Rana catesbeiana*) on native Ranid frogs in Oregon. Copeia.

[b36] Pearl CA, Adams MJ, Wente WH (2007). Characteristics of Columbia spotted frog (*Rana luteiventris*) oviposition sites in northeastern Oregon, USA. West. N. Am. Nat.

[b37] Pearl CA, Adams MJ, Leuthold N (2009a). Breeding habitat and local population size of the Oregon spotted frog (*Rana pretiosa*) in Oregon, USA. Northwest Nat.

[b38] Pearl CA, Bowerman J, Adams MJ, Chelgren ND (2009b). Widespread occurrence of the chytrid fungus *Batrachochytrium dendrobatidis* on Oregon spotted frogs (*Rana pretiosa*. EcoHealth.

[b39] Pearl CA, Adams MJ, Bury RB, Wente WH, McCreary B (2009c). Evaluating amphibian declines with site revisits and occupancy models: status of montane anurans in the pacific northwest USA. Diversity.

[b400] Pilliod DS, Arkle RS, Robertson JM, Murphy MA, Funk WC Effects of changing climate on aquatic habitat and connectivity for remnent populations of a wide-ranging frog species in an arid landscape. Ecol. Evol.

[b40] Pilliod DS, Peterson CR (2001). Local and landscape effects of introduced trout on amphibians in historically fishless watersheds. Ecosystems.

[b41] Pilliod DS, Scherer RD (2015). Managing habitat to slow or reverse population declines of the Columbia spotted frog in the northern Great Basin. J. Wildl. Manag.

[b42] Pilliod DS, Peterson CR, Ritson PI (2002). Seasonal migration of Columbia spotted frogs (*Rana luteiventris*) among complementary resources in a high mountain basin. Can. J. Zool.

[b43] Pilliod DS, Hossack BR, Bahls PF, Bull EL, Corn PS, Hokit G (2010a). Non-native salmonids affect amphibian occupancy at multiple spatial scales. Divers. Distrib.

[b44] Pilliod DS, Muths E, Scherer RD, Bartelt PE, Corn PS, Hossack BR (2010b). Effects of amphibian chytrid fungus on individual survival probability in wild boreal toads. Conserv. Biol.

[b45] Pilliod DS, Griffiths RA, Heatwole H, Wilkinson JW, Kuzmin SL (2012). Ecological impacts of non-native species. Conservation and decline of amphibians: ecological aspects, effect of humans, and management.

[b46] Pilliod DS, Arkle RS, Maxell BA (2013). Persistence and extirpation in invaded landscapes: patch characteristics and connectivity determine effects of non-native predatory fish on native salamanders. Biol. Invasion.

[b300] Pollock MM, Beechie TJ, Wheaton JM, Hordan CE, Bouwes N, Weber N, Volk C (2014). Using beaver dams to restore incised stream ecosystems. Bioscience.

[b47] Popescu VD, Gibbs JP (2009). Interactions between climate, beaver activity, and pond occupancy by the cold-adapted mink frog in New York State, USA. Biol. Conserv.

[b48] Reaser JK (1996). *Rana pretiosa* - vagility. Herpetol. Rev.

[b49] Ryan ME, Palen WJ, Adams MJ, Rochefort RM (2014). Amphibians in the climate vise: loss and restoration of resilience of montane wetland ecosystems in the western US. Front. Ecol. Environ.

[b50] Sepulveda AJ, Layhee M, Stagliano D, Chaffin J, Begley A, Maxell B (2014).

[b51] Service, U.S.F.A.W (2013).

[b52] Shelford VE (1931). Some concepts of bioecology. Ecology.

[b53] Stevens CE, Paszkowski CA, Foote AL (2007). Beaver (*Castor canadensis*) as a surrogate species for conserving anuran amphibians on boreal streams in Alberta, Canada. Biol. Conserv.

[b54] U.S. Environmental Protection Agency (USEPA) and the U.S. Geological Survey (USGS) (2012).

[b500] Van Horne R (2012).

[b55] Welch NE, MacMahon JA (2005). Identifying habitat variables important to the rare Columbia spotted frog in Utah (USA): an information-theoretic approach. Conserv. Biol.

[b56] Welsh HH, Hodgson GR (2011). Spatial relationships in a dendritic network: the herpetofaunal metacommunity of the Mattole River catchment of northwest California. Ecography.

[b57] Wente WH, Adams MJ, Pearl C (2005). Evidence of decline for Bufo boreas and *Rana luteiventris* in and around the northern Great Basin, western USA. Alytes.

[b58] Whittaker RH (1956). Vegetation of the great smoky mountains. Ecol. Monogr.

